# Glutamine, fish oil and antioxidants in critical illness: MetaPlus trial post hoc safety analysis

**DOI:** 10.1186/s13613-016-0220-y

**Published:** 2016-12-12

**Authors:** Zandrie Hofman, Sophie Swinkels, Arthur R. H. van Zanten

**Affiliations:** 1 Nutricia Research, Utrecht, The Netherlands; 2 Department of Intensive Care, Gelderse Vallei Hospital, Willy Brandtlaan 10, 6716 RP Ede, The Netherlands

**Keywords:** Critically ill, Mortality, Glutamine, Fish oil, Antioxidants, Nutritional support, Immune-modulating nutrients, Enteral nutrition, Clinical outcome

## Abstract

**Background:**

The role of plasma glutamine, fish oil and antioxidants concentrations in the treatment effect of immune-modulating high-protein versus high-protein enteral nutrition on 6-month mortality in critically ill patients is explored, as unexpected negative outcomes of recent large randomized controlled trials on immune-modulating nutrients have raised questions about safety of these interventions.

**Methods:**

Post hoc analysis of the MetaPlus randomized controlled trial which was performed in a total of 301 medical, surgical and trauma critically ill patients in fourteen European intensive care units. Patients received either immune-modulating (glutamine, fish oil and antioxidant enriched) high-protein (IMHP) or isocaloric high-protein (HP) enteral nutrition. Six-month mortality and baseline, day 4 and day 8 plasma concentrations of glutamine, (eicosapentaenoicacid + decosahexaenoicacid)/long-chain fatty acid plasma level ratio ((epa + dha)/lcf ratio), selenium, vitamin c, vitamin e and zinc were measured.

**Results:**

The harmful treatment effect of the IMHP versus HP enteral nutrition on 6-month mortality was only demonstrated in the medical subgroup (HR 2.52, 95% CI 1.36–4.78, *P* = 0.004). Among medical patients, when corrected for age groups and APACHE-II scores, there were no statistically significant associations between baseline plasma levels and 6-month mortality, except for zinc (HR 1.06, 95% CI 1.00–1.12, *P* = 0.026). IMHP feeding resulted in statistically significant increase in plasma levels of glutamine, vitamin e, vitamin c and (epa + dha)/lcf ratio from baseline to day 4, while only the change from baseline to day 4 of (epa + dha)/lcf ratio was statistically significant associated with 6-month mortality (HR 1.18, 95% CI 1.02–1.35, *P* = 0.021) and identified as mediator for the harmful treatment effect of IMHP enteral nutrition among medical ICU patients.

**Conclusion:**

We hypothesize that the harmful effect of IMHP compared to HP enteral nutrition in a heterogeneous group of critically ill patients is limited to the medical critically ill patients and mediated by an early increase in (epa + dha)/lcf ratio.

*Trial Registration* Dutch Trial Register 26 January 2010 (NTR2181 http://www.trialregister.nl/trialreg/admin/rctview.asp?TC=2181).

**Electronic supplementary material:**

The online version of this article (doi:10.1186/s13613-016-0220-y) contains supplementary material, which is available to authorized users.

## Background

Safety of immune-modulating nutrients like glutamine, fish oils and antioxidants to modulate infectious morbidity and enhance recovery from critical illness is under debate [1–6]. Recent large randomized trials in ICU patients failed to demonstrate benefits of immune-modulating nutrients and even demonstrated increased mortality [7–11].

Unexpected negative study results raised concern about safety of immune-modulating nutrients. It has been suggested that supplementation with immune-modulating nutrients should be reserved for specifically identified patients with compromised availability and plasma levels should be measured before supplementation [5]. However, low plasma levels could reflect adaptive and beneficial stress responses rather than conditional deficiency. The conditional deficiency hypothesis of glutamine has been challenged [12], and it has been suggested that interfering with a potential adaption could be deleterious [3].

This could suggest that supplementation with immune-modulating nutrients in critically ill patients does not improve outcome [3] and for safety reasons any patient with multi-organ failure in the ICU should not receive immune-modulating nutrients [2].

Studying mortality in critical illness and plasma status of immune-modulating nutrients after supplementation may provide pathophysiological insight and potentially facilitate redefinition of practice guidelines recommendations.

In the MetaPlus study, immune-modulating high-protein (IMHP) enteral feeding enriched with glutamine, fish oil and antioxidants was compared with standard high-protein (HP) enteral feeding in critically ill patients, and a hazard ratio (HR) of 1.57 (95% CI, 1.03–2.39; *P* = 0.04) was shown for 6-month mortality adjusted for age and APACHE-II score comparing IMHP with HP [7]. Univariate analysis suggested that this harmful effect was mainly observed among medical patients, although interaction between “type of patient” and “treatment” was not tested. The MetaPlus study is unique as plasma concentrations of glutamine, eicosapentaenoicacid + decosahexaenoicacid)/long-chain fatty acid plasma level ratio ((epa + dha)/lcf ratio), selenium, vitamin c, vitamin e and zinc were obtained at baseline (before treatment) and at days 4 and 8.

In this post hoc analysis, we address the interaction between IMPH versus HP and medical versus non-medical patients and 6-month mortality and subsequently associations of baseline plasma levels and changes in immune-modulating nutrient plasma levels and 6-month mortality.

## Materials and methods

Study design, methodology and population have been reported previously [7]. The MetaPlus trial was a prospective, randomized, multicenter, international, double-blind, controlled, parallel-group trial. In total, 301 adult mechanically ventilated ICU patients admitted to 14 participating ICUs in 4 European countries that were expected to be ventilated for >72 h and to require EN for >72 h were 1:1 randomized to IMHP or HP enteral nutrition, stratified per site and type of patient (medical, surgical non-trauma patients, trauma-surgical patients and trauma-non-surgical patients). Patients assigned to IMHP received a glutamine, omega-3 fatty acids and antioxidant (selenium, vitamin c, vitamin e and zinc)-enriched experimental tube feed. Those assigned to HP received an isocaloric/isonitrogenous high-protein tube feed (Nutrison Advanced Protison, NV Nutricia, Zoetermeer, The Netherlands). Patients were fed according to routine practice with recommendations toward early enteral feeding up to target energy requirements of 25 kcal/kg body weight with a maximum of 2500 kcal/day. Patients received study formulations during ICU stay for a maximum of 28 days.

The primary endpoint was incidence of new infections. Among others, 28 days and 6-month mortality were secondary parameters. Blood samples were taken at baseline, day 4 and day 8 for plasma glutamine, (epa + dha)/lcf ratio, selenium, vitamin c, vitamin e and zinc levels. Laboratory methods to measure plasma levels of immune-modulating nutrients are available in the Additional file 1: electronic supplement (S1).

Ethics committees and regulatory authorities approved the protocol and accompanying documents. The study protocol was registered in the Dutch Trial Register on January 26, 2010 (NTR2181, http://www.trialregister.nl/trialreg/admin/rctview.asp?TC=2181).

### Post hoc statistical analyses

In this post hoc analysis, Cox proportional hazard regression models were used to analyze the time until death with subjects still alive at 6 months after study entry treated as censored cases (the 6-month mortality). Interactions in these models were tested including an interaction term for interaction of “IMHP versus HP group” and subsequently “type of patient (medical vs. non-medical)”, “gender”, “age groups”, “APACHE-II”, “SOFA-score”, “baseline plasma glutamine”, “baseline plasma epa + dha/lcf ratio”, “baseline plasma selenium”, “baseline plasma zinc” and “baseline plasma vitamin e”. For interaction tests, the criterion was set a *P* value <0.10.

In case of statistical significant interaction, treatment effects on 6-month mortality were tested by Cox proportional hazard regression analysis with “age groups” and “Acute Physiology and Chronic Health Evaluation-II (APACHE-II) scores” as covariates within each subgroup. Associations of baseline plasma concentrations and 6-month mortality were tested using univariate Cox proportional hazard regression and multivariate Cox proportional hazard regression with “age groups” and “APACHE-II score” as covariates.

Differences between IMHP and HP in plasma concentration changes from baseline to day 4 and from baseline to day 8 were analyzed as continuous variables using two-sample *t* tests. The criterion was set at *P* value <0.05.

The relation between plasma nutrient status changes and 6-month mortality was evaluated with multivariate Cox proportional hazard models for 6-month mortality including “age groups”, “APACHE-II score” and “baseline plasma nutrient concentrations” as covariates.

**Fig. 1:**
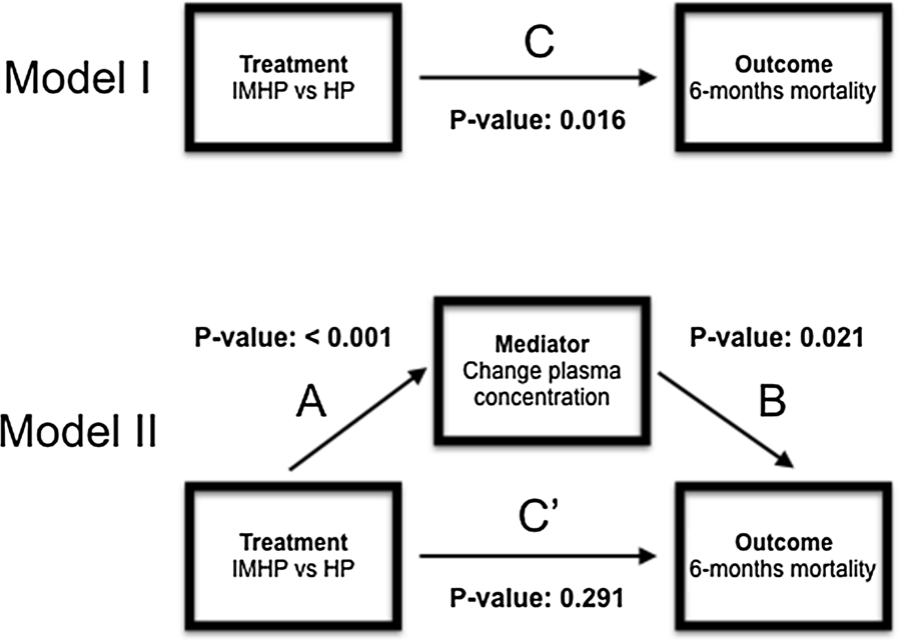
Illustration of causal step approach for changes in (epa + dha)/lcf plasma level ratio from baseline to day 4 as mediator for treatment effect. *IMHP* immune-modulating high-protein enteral nutrition, *HP* high-protein enteral nutrition, *A*, *B*, *C*, *C*′ association path. Method based on: Baron and Kenny [13]. For mediator analyses, it is important not only that a given treatment (IMHP vs. HP) is associated with the outcome (6-month mortality), but also that the treatment induces changes in mediator plasma concentrations (association path A) and that these changes are associated with the outcome (association path B). In case a potential mediator is inserted into multivariate analysis (Model II), the effect of the treatment (C′) should disappear. In other words, the treatment induces statistical significant changes in the outcome (6-month mortality increase) through an increase in plasma levels, these increases in plasma levels are associated with the increased 6-month mortality, and this effect is mediated through the treatment and not due to other factors

To assess which plasma nutrient changes mediated the effects on 6-month mortality, the causal steps approach was used [13]. This approach requires estimating each path depicted in Fig. 1.

Model I in Fig. 1 represents treatment effects of IMHP versus HP on 6-month mortality as analyzed using Cox proportional hazard regression analysis with “age groups” and “APACHE-II score” as covariates. In Model II, changes in plasma concentrations are suggested to mediate the relationship between treatment (IMHP vs. HP) and outcome (6-month mortality) when both the A and B association paths are statistically significant present and the C′ path association coefficient is closer to zero than in the C path in model I.

In Model II, the A path depicts the relation between the intervention (IMHP vs. HP) and changes in plasma nutrient status. This was analyzed using two-sample *t* tests. The B path describes the relation between changes in plasma nutrient status and 6-month mortality and analyzed using multivariate Cox proportional hazard models for 6-month mortality including “age groups”, “APACHE-II score” and “baseline plasma nutrient concentrations” as covariates. The Cox proportional hazard regression model with “age groups” and “APACHE-II score” as covariates was used to evaluate the C and C’ path, only for potential mediators having significant (*P* < 0.05) associations in both the A and B paths. The C’ path is evaluated in a Cox proportional hazard regression model including changes in plasma nutrient status variables as possible mediators.

Statistical analyses were performed on an intention-to-treat basis with SAS software, version 9.2 (SAS Institute Inc, Cary, USA).

## Results

“Type of patient (medical vs. non-medical)” was the only factor that interacted with the effect of “treatment group (IMHP vs. HP)” (interaction term *P* = 0.051). Table 1 a/b depicts the IMHP versus HP treatment effects among medical and non-medical patients, showing a statistically significant treatment effect among medical patients with a HR of 2.52 (95% CI 1.36–4.78, *P* = 0.004) and no treatment effect in the non-medical group with an HR of 0.97 (95% CI 0.54–1.74, *P* = 0.909).

**Table 1:** Multivariate Cox proportional hazard regression analysis on treatment effects among medical patients (a) and non-medical patients (b)

Parameter	Parameter estimate	Standard error	Hazard ratio	95% CI of the hazard ratio	*P* value
*1a: Medical patients (n* = *109)*					
IMHP versus HP	0.922	0.319	2.515	[1.360, 4.783]	0.004
Age group 1 versus 4	−2.651	0.788	0.071	[0.011, 0.275]	<0.001
Age group 2 versus 4	−1.439	0.393	0.237	[0.110, 0.522]	<0.001
Age group 3 versus 4	−1.120	0.409	0.326	[0.145, 0.733]	0.006
APACHE-II score	0.017	0.019	1.017	[0.980, 1.057]	0.376
*2a: Non*-*medical patients (n* = *192)*					
IMHP versus HP	−0.034	0.296	0.967	[0.540, 1.736]	0.909
Age group 1 versus 4	−1.595	0.533	0.203	[0.068, 0.562]	0.003
Age group 2 versus 4	−1.489	0.434	0.226	[0.095, 0.531]	<0.001
Age group 3 versus 4	−0.467	0.381	0.627	[0.299, 1.353]	0.219
APACHE-II score	0.101	0.026	1.106	[1.052, 1.165]	<0.001

*IMHP* immune-modulating high-protein enteral nutrition, *HP* high-protein enteral nutrition, *age group 1* age ≤50 year, *age group 2* age 51–70 years, *age group 3* age 71–80 years, *age group 4* age >80 years, *APACHE-II* acute physiology and chronic health evaluation-II

Associations between baseline plasma concentrations and 6-month mortality in all patients and in medical and non-medical subgroups are shown in Table 2. Univariate analyses showed statistically significant associations between baseline glutamine and 6-month mortality and between (epa + dha)/lcf ratio and 6-month mortality. Baseline zinc plasma concentrations were statistically significant associated with 6-month mortality in non-medical patients. However, when corrected for “age groups” and “APACHE-II scores,” there were no statistically significant associations, except for baseline zinc concentration and 6-month mortality among medical patients.

**Table 2:** Associations of baseline plasma concentrations of immune-modulating ingredients and 6-month mortality in all, medical and non-medical critically ill patients

Immunonutrient (unit)	Univariate analysis					Multivariate analysis				
	Coef.	SE	Hazard ratio	95% CI of the hazard ratio	*P* value	Coef.	SE	Hazard ratio	95% CI of the hazard ratio	*P* value
*2a: All patients (n* *=* *301)*										
Glutamine (μmol/L)	0.00119	0.00059	1.001	[1.000, 1.002]	0.046	0.00034	0.00065	1.000	[0.999, 1.001]	0.599
(epa + dha)/lcf ratio (×10^−2^)	0.21174	0.09876	1.236	[1.012, 1.491]	0.032	−0.02082	0.10415	0.979	[0.794, 1.195]	0.842
Selenium (μmol/L)	0.06623	0.16564	1.068	[0.737, 1.416]	0.689	0.11961	0.14465	1.127	[0.814, 1.448]	0.408
Vit E (μmol/L)	−0.00416	0.01402	0.996	[0.968, 1.023]	0.766	−0.00750	0.01458	0.993	[0.964, 1.021]	0.607
Vit C (μmol/L)	0.00297	0.00754	1.003	[0.986, 1.015]	0.694	−0.00507	0.00817	0.995	[0.977, 1.009]	0.535
Zinc (μmol/L)	0.01189	0.02532	1.012	[0.961, 1.061]	0.639	0.02327	0.02437	1.024	[0.974, 1.071]	0.340
*2b: Medical patients (n* *=* *109)*										
Glutamine (μmol/L)	−0.00011	0.00090	1.000	[0.998, 1.002]	0.901	−0.00025	0.00095	1.000	[0.998, 1.002]	0.792
(epa + dha)/lcf ratio (×10^−2^)	0.16155	0.12798	1.175	[0.905, 1.496]	0.207	−0.04175	0.13716	0.959	[0.727, 1.246]	0.761
Selenium (μmol/L)	0.06647	0.17729	1.069	[0.689, 1.420]	0.708	0.06778	0.18363	1.070	[0.685, 1.457]	0.712
Vit E (μmol/L)	−0.00370	0.01981	0.996	[0.957, 1.035]	0.852	−0.00299	0.02107	0.997	[0.955, 1.038]	0.887
Vit C (μmol/L)	−0.00611	0.00966	0.994	[0.972, 1.009]	0.527	−0.00773	0.00995	0.992	[0.970, 1.008]	0.437
Zinc (μmol/L)	0.04600	0.02592	1.047	[0.992, 1.098]	0.076	0.06078	0.02729	1.063	[1.004, 1.118]	0.026
*2c: Non-medical patients*										
Glutamine (μmol/L)	0.00169	0.00074	1.002	[1.000, 1.003]	0.023	0.00067	0.00076	1.001	[0.999, 1.002]	0.377
(epa + dha)/lcf ratio (×10^−2^)	0.29408	0.14469	1.342	[0.996, 1.757]	0.042	0.03667	0.16425	1.037	[0.740, 1.410]	0.823
Selenium (μmol/L)	0.06196	0.29028	1.064	[0.570, 1.785]	0.831	0.11013	0.27021	1.116	[0.625, 1.816]	0.684
Vit E (μmol/L)	−0.01053	0.02043	0.990	[0.948, 1.027]	0.606	−0.01162	0.02052	0.988	[0.949, 1.028]	0.571
Vit C (μmol/L)	0.02023	0.01849	1.020	[0.982, 1.056]	0.274	0.01667	0.01899	1.017	[0.978, 1.054]	0.380
Zinc (μmol/L)	−0.10003	0.04612	0.905	[0.824, 0.988]	0.030	−0.06168	0.04475	0.940	[0.857, 1.022]	0.168

The multivariate statistical model includes the variables “age group” and “Apache-II score” as covariates. Coef = coefficient. The coefficient is the Cox proportional hazard regression parameter estimate; a positive coefficient indicates a worse prognosis, and a negative coefficient indicates a protective effect of the variable on 6-month mortality. Coefficient values represent changes per unit of nutrient for the different immunonutrients and change per percentage for (epa + dha)/lcf ratio. SE is the parameter estimate standard regression. *P* values represent Chi-square statistic testing the null hypothesis that the estimate is zero

**Fig. 2:**
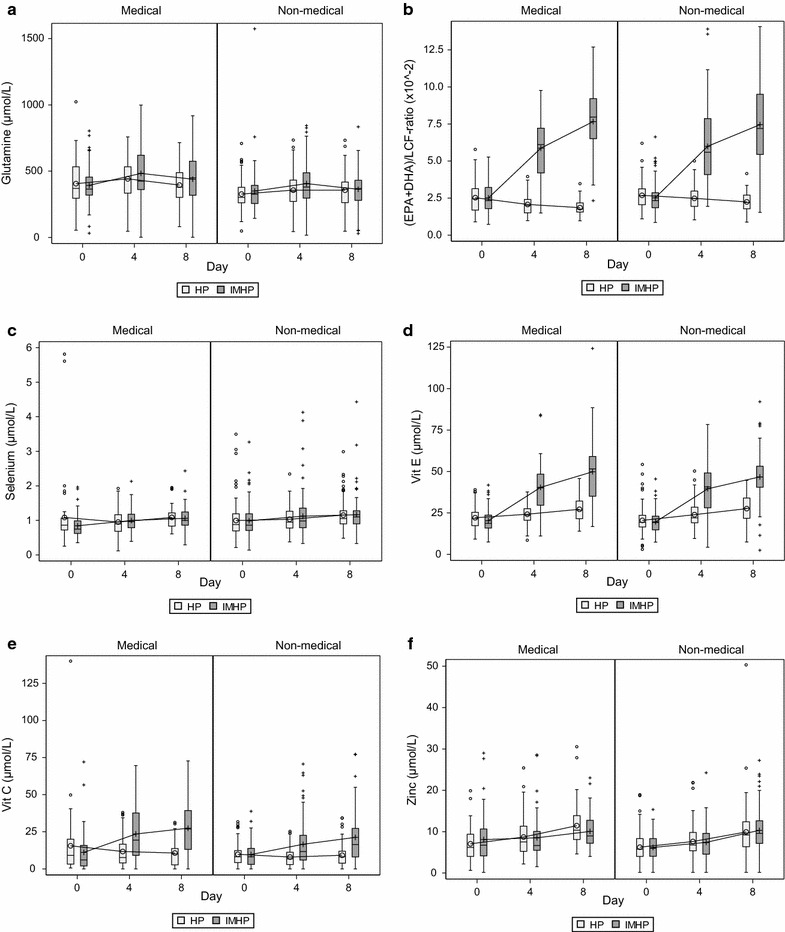
Boxplot figures representing the plasma concentration values and variations of the immune-modulating nutrients at baseline, day 4 and day 8 among medical and non-medical patients. **a** Glutamine. **b** (epa + dha)/lcf ratio. **c** Selenium. **d** Vitamin e. **e** Vitamin c. **f** Zinc. *IMHP* immune-modulating high-protein enteral nutrition, *HP* high-protein enteral nutrition. Boxplot interpretation: 0 or +: average value, −: median, rectangle bottom: quartile 1 cutpoint (25th percentile), rectangle upper: quartile 3 cutpoint (75th percentile). 0 or +: outliers more than 1.5 times inter quartile range above quartile 3 or below quartile 1, T: highest or lowest level not being an outlier

Baseline plasma concentrations and plasma concentrations on day 4 and day 8 for IMHP and HP are shown in Fig. 2 for the medical and non-medical patients. There were no statistical significant differences in baseline concentrations between IMHP and HP groups. In medical and non-medical patients, the changes in plasma levels from baseline to day 4 and from baseline to day 8 were statistically significant larger in IMHP-treated patients compared with HP patients for (epa + dha)/lcf ratio, vitamin e and vitamin c, while plasma glutamine change from baseline to day 4 was statistically significant in the medical patients (Table 3).

**Table 3:** Baseline plasma concentrations of immune-modulating ingredients and changes from baseline on day 4 and 8 in medical and non-medical critically ill patients

Immune-modulating nutrient concentrations	IMHP (*n* = 54) mean (SD)	HP (*n* = 55) mean (SD)	*P* value
*3a: Medical patients (n* *=* *109)*			
Glutamine (μmol/L)			
Baseline (BL)	393 (155)	406 (168)	0.687
Day 4—BL	102 (141)	36 (141)	0.017
Day 8—BL	77 (148)	10 (168)	0.070
(epa + dha)/lcf ratio (×10^−2^)			
Baseline	2.5 (1.1)	2.5 (1.1)	0.948
Day 4—BL	3.3 (2.1)	−0.4 (0.5)	<0.001
Day 8—BL	5.3 (2.2)	−0.7 (0.7)	<0.001
Selenium (μmol/L)			
Baseline	0.84 (0.37)	1.08 (1.00)	0.113
Day 4—BL	0.16 (0.33)	−0.16 (1.08)	0.057
Day 8—BL	0.21 (0.43)	−0.08 (1.26)	0.200
Vit E (μmol/L)			
Baseline	20.3 (7.6)	22.1 (7.2)	0.208
Day 4—BL	19.5 (13.9)	2.5 (6.3)	<0.001
Day 8—BL	29.1 (18.8)	5.7 (8.9)	<0.001
Vit C (μmol/L)			
Baseline	11.1 (14.0)	15.6 (22.0)	0.245
Day 4—BL	13.6 (17.5)	−4.4 (21.9)	<0.001
Day 8—BL	19.7 (18.5)	−5.6 (26.3)	<0.001
Zinc (μmol/L)			
Baseline	8.09 (6.05)	7.06 (4.05)	0.321
Day 4—BL	0.73 (4.17)	1.56 (3.56)	0.308
Day 8—BL	2.09 (3.90)	3.66 (4.34)	0.119

Immune-modulating nutrient concentrations	IMHP (*n* = 98) mean (SD)	HP (*n* = 94) mean (SD)	*P* value
*3b Non-medical patients (n* *=* *192)*			
Glutamine (μmol/L)			
Baseline (BL)	350 (163)	327 (104)	0.252
Day 4—BL	55 (180)	27 (118)	0.206
Day 8—BL	16 (191)	30 (137)	0.586
(epa + dha)/lcf ratio (×10^−2^)			
Baseline	2.5 (1.0)	2.7 (0.9)	0.226
Day 4—BL	3.5 (2.2)	−0.2 (0.6)	<0.001
Day 8—BL	5.0 (2.7)	−0.4 (0.6)	<0.001
Selenium (μmol/L)			
Baseline	0.99 (0.46)	0.99 (0.55)	0.928
Day 4—BL	0.13 (0.59)	0.03 (0.56)	0.272
Day 8—BL	0.17 (0.73)	0.15 (0.68)	0.900
Vit E (μmol/L)			
Baseline	19.6 (6.6)	20.5 (8.2)	0.399
Day 4—BL	19.7 (13.9)	3.3 (5.8)	<0.001
Day 8—BL	26.7 (14.4)	6.6 (7.5)	<0.001
Vit C (μmol/L)			
Baseline	9.7 (8.2)	9.8 (7.7)	0.904
Day 4—BL	7.0 (13.2)	−2.1 (6.3)	<0.001
Day 8—BL	11.6 (14.7)	−0.2 (9.3)	<0.001
Zinc (μmol/L)			
Baseline	6.18 (3.25)	6.24 (3.76)	0.907
Day 4—BL	1.20 (3.13)	1.36 (3.85)	0.754
Day 8—BL	3.98 (4.59)	3.44 (6.39)	0.538

All tests were performed with two-sample *t* tests

*IMHP* immune-modulating high-protein enteral nutrition, *HP* high-protein enteral nutrition, *sd* standard deviation, *(epa* *+* *dha)/lcf ratio* (eicosapentaenoicacid + decosahexaenoicacid)/long-chain fatty acid plasma level ratio, *BL* baseline, *Day 4—BL* change in plasma concentration from baseline to day 4, *Day 8—BL* change in plasma concentration from baseline to day 8

The associations between changes in plasma concentrations from baseline to day 4 and day 8 and 6-month mortality are shown in Table 4. There is a statistically significant positive association of changes in (epa + dha)/lcf ratios from baseline to day 4 with 6-month mortality in the medical patients, and a statistically significant negative association of changes in zinc from baseline to day 8 with 6-month mortality in the non-medical patients.

**Table 4:** Associations of changes in plasma concentrations from baseline to day 4 and day 8 with 6-month mortality in medical and non-medical critically ill patients

Immunonutrient	Baseline to day 4					Baseline to day 8				
	Coef.	SE	Hazard ratio	95% CI of the hazard ratio	*P* value	Coef.	SE	Hazard ratio	95% CI of the hazard ratio	*P* value
*4a: Medical patients*										
Glutamine (μmol/L)	−0.002	0.001	0.998	[0.996, 1.000]	0.111	−0.001	0.001	0.999	[0.996, 1.001]	0.302
(epa + dha)/lcf ratio (×10^−2^)	0.162	0.070	1.176	[1.023, 1.348]	0.021	0.055	0.053	1.057	[0.949, 1.170]	0.294
Selenium (μmol/L)	0.487	0.457	1.628	[0.644, 3.892]	0.286	−0.551	0.615	0.576	[0.159, 1.776]	0.370
Vit E (μmol/L)	−0.005	0.015	0.995	[0.964, 1.024]	0.758	0.009	0.012	1.009	[0.985, 1.031]	0.446
Vit C (μmol/L)	−0.006	0.011	0.994	[0.971, 1.016]	0.614	−0.001	0.011	0.999	[0.976, 1.020]	0.944
Zinc (μmol/L)	−0.013	0.049	0.988	[0.890, 1.080]	0.799	−0.093	0.064	0.912	[0.794, 1.020]	0.145
*4b: Non*-*medical patients*										
Glutamine (μmol/L)	0.001	0.001	1.001	[0.999, 1.003]	0.439	0.002	0.001	1.002	[0.999, 1.004]	0.180
(epa + dha)/lcf ratio (×10^−2^)	−0.015	0.057	0.985	[0.876, 1.095]	0.793	−0.040	0.050	0.961	[0.866, 1.057]	0.431
Selenium (μmol/L)	0.353	0.359	1.424	[0.675, 2.807]	0.324	−0.530	0.440	0.589	[0.238, 1.326]	0.228
Vit E (μmol/L)	−0.010	0.013	0.990	[0.965, 1.014]	0.429	−0.005	0.012	0.995	[0.971, 1.018]	0.667
Vit C (μmol/L)	−0.005	0.014	0.995	[0.968, 1.021]	0.700	−0.009	0.013	0.991	[0.966, 1.016]	0.499
Zinc (μmol/L)	−0.028	0.053	0.973	[0.873, 1.074]	0.600	−0.102	0.051	0.903	[0.812, 0.991]	0.045

The coefficient is the Cox proportional hazard regression parameter estimate; a positive coefficient indicates a worse prognosis and a negative coefficient indicates a protective effect of the variable on 6-month mortality. Coefficient values represent changes per unit of nutrient for the different immunonutrients and change per percentage for (epa + dha)/lcf ratio. SE is the parameter estimate standard regression. *CI* confidence interval. *P* values represent Chi-square statistic testing the null hypothesis that the estimate is zero

The change in (epa + dha)/lcf ratios from baseline to day 4 is a potential candidate for mediator analysis as for this parameter IMHP versus HP conferred a statistically significant increase in plasma concentrations (Table 3; Fig. 1 association path A) and a statistically significant association between plasma concentration changes and 6-month mortality (Table 4; Fig. 1 association path B). Mediator analysis showed that a change in (epa + dha)/lcf ratio from baseline to day 4 was found to be a mediator for the treatment effect of IMHP versus HP concerning 6-month mortality, as including change in (epa + dha)/lcf ratio from baseline to day 4 into the statistical model decreased the coefficient of the statistical treatment effect on 6-month mortality (coefficient 0.823; *P* value: 0.016) to a non-statistical significant treatment effect (coefficient 0.618; *P* value: 0.291, Fig. 1).

Evaluation of the presence of A and B association paths in model II Fig. 1 was based on the criterion *P* value < 0.05. Relaxing this criterion to a *P* value < 0.15 did result in more parameters being investigated as possible mediators. However, none of those showed any suggestion for a mediating effect (data not shown).

Additional analysis on the association between the mediator (epa + dha)/lcf ratio and 6-month mortality showed that patients with an increase (≥0) in (epa + dha)/lcf plasma level ratios compared with those with a decrease (<0) from baseline to day 4 demonstrated higher 6-month mortality risk (*P* = 0.007, HR = 2.8) (Table 5).

**Table 5:** Associations between changes in (epa + dha)/lcf ratio from baseline to day 4 and 6-month mortality in medical critically ill patients

(epa + dha)/lcf ratio parameters	6-months mortality		Cox proportional hazard model analysis				
	Incidence (%)		Coef.	SE	Hazard ratio	95% CI of the hazard ratio	*P* value
(epa + dha)/lcf ratio (×10^−2^), continuous			0.162	0.070	1.176	[1.023, 1.348]	0.021
(epa + dha)/lcf ratio (×10^−2^), recoded to quartiles							
Q1 cutpoint: <−0.44	33	Q1 versus Q4	−0.667	0.486	0.513	[0.191, 1.324]	0.170
Q2 cutpoints: ≥−0.44 to <0.11	35	Q2 versus Q4	−0.960	0.506	0.383	[0.137, 1.023]	0.058
Q3 cutpoints: ≥0.11 to <3.19	48	Q3 versus Q4	0.076	0.447	1.079	[0.447, 2.627]	0.865
Q4 cutpoint: ≥3.19	46						
		Overall					0.105
(epa + dha)/lcf ratio (×10^−2^), recoded to ≥0 versus <0							
<0	34	≥ 0 versus <0	1.030	0.383	2.800	[1.344, 6.098]	0.007
≥0	46						

*Coef* coefficient. The coefficient is the Cox proportional hazard regression parameter estimate; a positive coefficient indicates a worse prognosis, and a negative coefficient indicates a protective effect of the variable on 6-month mortality. Coefficient values represent changes per percentage for (epa + dha)/lcf ratio. SE is the parameter estimate standard regression. *P* values represent Chi-square statistic testing the null hypothesis that the estimate is zero

## Discussion

Based on this post hoc analysis of the MetaPlus prospective randomized double-blind multicenter trial in which a high-protein enteral nutrition enriched with immune-modulating nutrients was compared with a standard isocaloric isonitrogenous high-protein enteral nutrition in a heterogeneous mechanically ventilated ICU population, we hypothesize that the harmful treatment effect of IMHP versus HP on 6-month mortality is specific for medical patients and that this harmful effect is mediated by an acute increase from baseline to day 4 in plasma (epa + dha)/lcf ratios as: (1) IMHP versus HP treatment results in a statistically significant increase in plasma (epa + dha)/lcf ratios at day 4, (2) an increase in (epa + dha)/lcf ratios was shown to be associated with 6-month mortality, and (3) including changes in (epa + dha)/lcf ratios from baseline to day 4 into the statistical model decreased the coefficient of the statistical treatment effect on 6-month mortality to a non-statistical significant treatment effect.

### Medical versus non-medical patients

The debate regarding safety of supplementation of immune-modulating nutrients has mainly emerged from negative results of the REDOXs [9] and OMEGA [10] trials with, respectively, 80 and 85% medical ICU patients, while in the SIGNET trial [8], showing no benefit nor harm of immune-modulating nutrients, only 25% of included patients were medical. Meta-analyses of results of immune-modulating nutrient intervention studies among surgical patients have not reported any harmful effects and even show positive effects [14, 15]. Our post hoc analyses suggest harmful treatment effects of IMHP on 6-month mortality among medical patients and not among non-medical patients, and are in line with these observations. Medical patients were older, had higher APACHE-II scores and more frequently suffered from sepsis and pulmonary diseases [7]. Previously, it was recommended not to use arginine in ICU patients [16] or patients with severe sepsis [17] due to possible vasodilatory effects from NO production in patients with circulatory shock. This mechanism might have played a role as glutamine serves as precursor for de novo arginine production through the citrulline–arginine pathway [18].

### Consequences for the glutamine debate

Our results show positive associations between higher baseline plasma glutamine and increased 6-month mortality in all patients and in non-medical patients. These findings contradict the hypothesis that glutamine is a conditional essential amino acid during critical illness [5].

This hypothesis is mainly based on one study from 2001 showing that ICU patients with plasma glutamine levels below 420 µmol/L show increased hospital mortality compared with patients with higher levels (420 µmol/L) [19]. However, mean age in the low glutamine group (74 years) was statistically significant different compared to the high glutamine group (63 years). Age was not included in the hospital mortality logistic regression analysis as separate explanatory factor. Including age potentially could have changed the strength and direction of associations, as in the MetaPlus study we showed that age is a strong independent mortality predictor [7] and our present analysis demonstrates that including age group covariates markedly influences associations between baseline levels and 6-month mortality. Recently, it was shown that not only low ICU admission plasma glutamine levels (<420 µmol/L), but also high plasma glutamine levels (>930 µmol/L) were associated with increased 6-month mortality, suggesting a U-shaped relation between plasma glutamine and 6-month mortality [20]. Furthermore, in septic patients, non-survivors had statistically significant higher median plasma glutamine levels (648 µmol/L) compared to survivors (460 µmol/L) [21]. In the MetaPlus study, only 2 patients had plasma levels >930 µmol/L and 95% confidence intervals of baseline plasma glutamine levels were 339–391 µmol/L (IMHP group) and 334–378 µmol/L (HP group)[7], suggesting the univariate positive association between baseline glutamine concentration and 6-month mortality is not influenced markedly by very high baseline concentrations.

The REDOXs trial showed increased mortality with glutamine supplementation and raised questions whether critical illness is associated with low plasma glutamine levels at all [2] and whether interfering with glutamine supplementation could be deleterious [3].

Our post hoc analysis does not show associations between changes in plasma glutamine and 6-month mortality. Moreover, changes in plasma glutamine concentrations were not found to be a mediator of harmful treatment effects of IMHP versus HP in medical patients. Therefore, our post hoc analyses do not support the hypothesis that interfering with glutamine supplementation in medical critically ill patients is deleterious. However, this should be interpreted with caution and it may not be concluded that glutamine is not harmful as the enteral dose of glutamine supplementation and the effect of IMHP on glutamine levels were very modest. Furthermore, plasma glutamine levels, independent of the treatment allocation, were very comparable in both groups as seen in Fig. 2.

### Consequences for the epa and dha debate

This post hoc analysis is the first to suggest that a change from baseline to day 4 in (epa + dha)/lcf plasma level ratios has a positive association with 6-month mortality risk. Average plasma (epa + dha)/lcf ratios among healthy persons across 16 European regions vary from 3.4 to 8.9%, due to differences in food intake [22]. In our study, average ICU admission values were below this range (2.5%), while after supplementation with IMHP for 4 or 8 days, averages were within these ranges. Among HP supplemented patients receiving no epa or dha, averages remained low, or even slightly decreased, suggesting a fast response of plasma status on epa and dha supplementation in IMHP patients. In the OMEGA study among acute lung injury ICU patients, Rice showed fast increases in plasma status within 3 days in epa, dha and gamma-linolenic-supplemented patients [10]. Rice concluded that fish oil supplementation might be harmful as illustrated by higher risk of 60-day mortality. Critics of the OMEGA trial suggested that twice daily bolus administration and higher protein intake in control patients confounded results [4]. However, our present results suggest that increased 6-month mortality in the IMHP group versus the HP group with similar amounts of protein was mediated through increases in (epa + dha)/lcf ratios from baseline to day 4 during continuous feeding. Therefore, without the perceived limitations of the OMEGA trial, we now report similar hazardous effects of omega-3 fatty acids supplementation among medical critically ill patients. Consequently, our findings do not support suggested clinical benefits of omega-3 fatty acids in enteral nutrition to preserve immune function and prevent aspects of the inflammatory response [23], as we did not observe any benefits on infectious morbidity [7]. As we did not measure biomarkers of immune function or inflammation, we cannot rule out that immune stimulation or anti-inflammatory effects have occurred. However, in the OMEGA trial no reductions in levels of inflammatory biomarkers despite marked increases in plasma omega-3 fatty acids were observed. The mechanism why increases in plasma omega-3 fatty acids are associated with increased mortality in medical critically ill patients remains unclear. We speculate that due to the suggested anti-inflammatory effect of fish oil IMHP not only reduced the systemic inflammatory response but also enhanced the compensatory anti-inflammatory response syndrome (CARS). It has become apparent that CARS is not simply a consecutive response to SIRS and hyperinflammation, but that both responses may occur simultaneously in the early phase after ICU admission. Possibly, we have induced the so-called persistent inflammatory immunosuppressed catabolic syndrome (PICS) in these patients [24]. Another speculation is based on interesting data on omega-3 fatty acids supplementation during exercise in healthy volunteers showing reduced maximal power output by 10% and maximal heart rate by 6% within 1–3 days of supplementation [25]. These negative cardiovascular and metabolic effects of omega-3 fatty acids supplementation during exercise probably are mediated by other mechanisms than omega-3 fatty acids incorporation into plasma membranes [25].

### Consequences for the antioxidant debate

Recent guidelines on nutritional support for critically ill patients by the Society of Critical Care Medicine and American Society of Parenteral and Enteral Nutrition indicate very low evidence for routine supplementation of antioxidants summarizing studies until 2013 [26]. In these guidelines, it is indicated that antioxidant and trace element supplementation is associated with significant reductions in overall mortality. Recent REDOXs and Signet trials did not show any benefit or harm from antioxidant supplementation, but a recent retrospective study on selenium supplementation in postoperative ICU patients with sepsis showed increased mortality in univariate analysis [11]. Our post hoc analyses show positive associations with baseline zinc concentrations and 6-month mortality in medical patients. However, there was no effect of IMHP versus HP treatment on plasma zinc levels and no association between increase in plasma zinc concentrations and 6-month mortality, with similar results for selenium, vitamin e and vitamin c. Therefore, we hypothesize that harmful effects of IMHP versus HP treatment is not due to these antioxidants.

To the best of our knowledge, the Metaplus study provides the largest database to study effects of nutritional treatment on plasma concentrations of immune-modulating nutrients in a heterogeneous mechanically ventilated ICU population. Although our data suggest mediator effects of changes in plasma (epa + dha)/lcf ratio, we must consider that findings are based on results from unplanned post hoc analyses. Furthermore, the relationship could be associative without direct causality if an underlying unknown confounding factor is involved. The intervention was a cocktail of various immune-modulating nutrients and may confer positive or negative effects on mortality, with or without interaction. Furthermore, post hoc analyses only generate hypotheses and therefore preclude firm recommendations.

## Conclusions

We hypothesize that the harmful effect of immune-modulating high-protein enteral nutrition compared to high-protein enteral nutrition in the MetaPlus trial studying a heterogeneous group of critically ill patients is limited to the medical critically ill patients and mediated by an early increase in (eicosapentaenoicacid + decosahexaenoicacid)/long-chain fatty acid plasma ratio, resulting in increased 6-month mortality.

## Additional file

**Additional file 1.** Immune-modulating nutrient analysis methods in plasma.
