# Supplementary material for: Glutamine, fish oil and antioxidants in critical illness: MetaPlus trial post hoc safety analysis
Source: Ann Intensive Care. 2016 Dec 12;6:119. doi: 10.1186/s13613-016-0220-y (PMC5153384; doi:10.1186/s13613-016-0220-y)
Supplement: Supplementary file 1 — Additional file 1. Immune-modulating nutrient analysis methods in plasma. [file 13613_2016_220_MOESM1_ESM.docx]

**S1 Electronic Supplement**

**Laboratory details of methods to measure plasma levels of immune-modulating nutrients**

Glutamine was analyzed after precipitation of proteins and polypeptides with perchloric acid and centrifugation. Content of the individual amino acids was determined by Ultra-Fast Liquid Chromatography (UFLC) using a pre-column derivatization with 0-phtaldialdehyde and fluorimetry as detection. Epa, dha and total fatty acids content were analyzed in plasma after extracting lipids from plasma and methylation. Fatty acid composition was quantitatively analyzed using gas chromatography. Epa and dha status was expressed as the total of epa and dha concentrations related to the total long chain fatty acids plasma levels ((epa+dha)/lcf-ratio). The content of vitamin c was measured in plasma supernatant after protein precipitation and centrifugation by UFCL using Ultraviolet absorbance as detection method. After protein precipitation and centrifugation supernatant was mixed with ethanol and again centrifugated. In this centrifugate, vitamin e was determined by UFLC using fluorometric properties for detection compared with standard solutions. Selenium was measured in plasma using Atomic Absorption Spectrometry and zinc was measured in plasma using Inductive Coupled Plasma-Optical Emission Spectrometry.
